# A New Word on "Taking the Bite."

**Published:** 1904-04

**Authors:** 


### A NEW WORD ON "TAKING THE BITE."

In writing on the subject of a "bite," I realize that the first question in the minds of most dentists will be, What now can be said on that worn-out subject?

I know the process of taking a bite did not originate in my office; so far as I am aware, however, the method I here present is original. It consists simply in causing the patient to manipulate the tongue in such a manner as to get the mind completely off the process of biting.

Prepare the wax and base-plate in the usual manner. Instruct the patient to apply the tongue to the roof of the mouth as far back as possible. Allow him to do this several times, opening and closing the mouth, before you insert the wax, and explain to him the great importance of keeping his tongue in that particular spot. Place the bite material in position and again give instructions regarding the patient's part in the process. Now tell him to bite into the wax, cautioning him frequently to keep the tongue in the correct position.

The above applies in case of "squash" bite or when the bite for a lower plate is being taken. In taking the bite for the upper plate or for upper and lower, using the base-plate method, I instruct the patient to place the tongue on the extreme heel of the plate instead of in the roof of the mouth, and explain the absolute necessity of holding it tightly against the palate.

With the tongue in this position, and the patient's mind on the tongue, it is practically impossible for the lower jaw to be thrown forward or laterally out of the correct relation with the upper.

Another advantage of this method is in taking a bite for the edentulous lower jaw. The tongue is out of the way and does not interfere with or move the lower base-plate and the operator may feel sure that the plate is resting correctly while the bite is being taken.

I have used this idea for the last two years with entire success, and am sure those who may try the method will be equally succssful, providing they sufficiently impress patients with the importance of keeping the tongue in position.—*Dr. R. E. Luther, Cosmos.*

---

To repair the engine cord take a piece of hollow shoe string, bring the ends together inside it and sew.—*Register.*

---

A plug of cotton dipped in a saturated solution of antipyrine and camphophenique, packed into alveoli after extracting, is good treatment for hemorrhage.—*Texas Journal.*

---

To Clean Vulcanite Files.—Apply chloroform and clean with stiff brush. This will remove all the rubber packed in trimming plates.—*Dr. W. H. Wheat, in Dental Hints.*

---

Re-Soldering.—To prevent the unsoldering or re-fusing of parts previously united, coat such surfaces with *crocus* (ferric peroxid) or a solution of *plumbago* or *whiting* in alcohol or water.—*Dr. H. J. Goslee, of Chicago, Ill., in Items of Interest.*

---

A New Obtundent.—Use Zinc-chloride in an alcoholic and chloroform solution instead of an aqueous one for the treatment of hypersensitive Dentin. The pain is greatly lessened, and it acts more rapidly, owing to the dessicating and obtunding effect of the chloroform and alcohol.—*Dr. R. H. Hofheinz, of Rochester, N. Y., in Dental Cosmos.*

---

To Remove Model From Articulator.—Grip the articulator in a bench vise and cut through the plaster with a small band-saw. Especially suitable for lower models, which are so easily broken. If sawn off at the right depth no paring is necessary before flasking.—*Dr. W. A. Brownlee, in Dominion Dental Journal.*

---

To Prevent Gagging and Retching From Insertion of Artificial Plates or Impressions.—Let the patient daily practice rubbing the roof of the mouth with the finger for a few weeks, and you will have success. —*Dr. H. S. Mann, of Saginaw, Mich., in Dental Office and Laboratory.*

---

To Remove Adhering Plaster From Vulcanite Plates.—Place the plate for a short time in water containing a small quantity of *Sulphate of Potassium.*—*Dr. A. F. Isham, of Buffalo, N. Y., in Dental Forum.*

---

Repairing Rubber Plates.—The old methods of dove-tail cutting, grooves, holes, etc., are entirely unnecessary. Cut out all the old rubber, and with a hot spatula pack in new against a clean-scraped surface.—*Dr. L. P. Haskell, of Chicago, Ill., in Dental Digest.*

---

To Remove Excess of Mercury in Amalgam Fillings.—Squeeze very dry that portion of amalgam that is left after filling, and hold it against the finished filling for a second; remove it, squeeze out and apply it again, until all excess of mercury is removed.—*Dr. W. B. Garrett, of Caruthersville, Mo., in Dental Era.*

---

To Assist in Gold Filling.—In many cases where a little thin coating of cement is used in lining a cavity the gold will adhere to the roughness made by the cement upon the walls of the cavity without any retaining pits.—*Dr. Gustavus North, Cedar Rapids, Ia.*

---

Plaster.—It is a well-known fact that plaster of paris is incapable of withstanding the application of extreme heat without marked change in bulk. It ought readily to be understood, therefore, that to undertake to cast base-plates or dentures to a plaster model, which necessarily must be subjected to heat in the process, invites failure of the plates fitting when placed in the mouth. To be brief, then, I would say that to cast a base-plate that will fit the mouth it must be cast to a model which is not perceptibly changed from perfect duplication of the form of the jaw upon which the plate is to rest.—*Dr. R. C. Brophy, Ex. Era.*

To Finish Amalgam Fillings.—Smooth your amalgam filling with a wet piece of Spunk. As it is without fibre, it produces a beautiful, smooth finish. Every dentist should have a little Spunk.—*Dr. Dodel.*

How to Mix Plaster.—Try mixing plaster by simply sifting into sufficient water. When you think that you have enough plaster, pour off the excess water and use *without stirring.* This produces a mix absolutely without bubbles.—*Dr. Dodel.*

A Handy Matrix for Melotte's Metal.—A piece of brass tubing one inch long, one inch in diameter, and 1/4 inch thick on one edge and tapering on the inside to 1/8 inch thick on the other edge, makes a very useful matrix for holding Melotte's metal dies. It is used exactly as the rubber ring that comes with a Melotte's outfit, only it is not removed from around the metal. By this method cracking a die is impossible.—*Dr. Dodel.*

Properties of the Capping Material.—A word about the properties of the "capping." The boracic acid is a refrigerant, permanent antiseptic, reducing inflammation; the creosote is a diffusible antiseptic and destroyer of gases; the zinc oxid is bland and non-conductive of thermal stimuli. The product formed by their admixture is so soft and velvety as to admit of its perfect adaptation without pressure.

With an accurate diagnosis and judicious use of this "capping" many pulps can be preserved that would be doomed to destruction.—*Dr. C. S. Smith.—Ex. Summary.*

Napkins.—Use a new napkin for every occasion, they are far preferable to a laundried one. One yard of eight cent bleached muslin will make thirty-six napkins six inches square, which can be made in about ten minutes. Tear the muslin and draw a few ravelings from each side, fold and press, and you have a nice fringed napkin.—*Dr. Gustavus North, Cedar Rapids, Iowa.*

To Assist in Holding Rubber Dam.—Where it is difficult to hold the rubber dam on the teeth, knot the floss-silk so a knot will appear on one or more surfaces of the teeth as the case may require, and it will hold the rubber in place.—*Dr. Gustavus North, Cedar Rapids, Iowa.*

Polishing Crowns.—To prevent marring a gold crown when polishing, wet the inside of the crown with a soap solution, fill it with modeling composition, and while the latter is still soft, thrust the end of a stick or instrument handle into it. When the crown is finished soften the composition in warm water and remove.—*Dr. B. H. Teague, of Augusta, Ga., in Dental Hints.*

In reference to the treatment of putrescent root canals, we treat them too much with medicine and do not pay enough attention to the mechanical part of the work. Very frequently a filling is inserted with strong drugs beneath, and the action of the drug or drugs is more direful than the putrescent condition we are trying to control.—*Dr. W. H. G. Logan, Review.*

Antikamia.—Antikamia is a valuable remedy for any pain that arises from nervous affections. Five grain tablets are a convenient form, and should be administered according to the condition of the patient. Dose one tablet, and if required follow with a tablet every hour until two or three have been taken, and relief will follow.—*Dr. Gustavus North, Cedar Rapids, Iowa.*

Low Fusing Porcelain Not Suitable for Contouring Work.—I consider it impractical to attempt contour filling with an inlay body that will not stand up as carved under a high heat. Low-fusing bodies have a tendency to assume a globular form when fused frequently. Then taking into consideration the great percentage of shrinkage—thirty-three and more per cent.—it is useless to waste words in regard to its usefulness, when we have good high-fusing bodies at our command in using which you do not have to contend with those objections. Suffice it to say that our best porcelain workers in this country advocate and use high-fusing porcelain bodies.—*Dr. D. O. M. LeCron, Dental Era.*

---

Refitting Lower Rubber Plates.—To refit a lower set of teeth when the plate is loose and the teeth a little short from the absorption of the tissue, place some soft wax in the groove of the lower plate, and place it in the mouth, have the patient close his mouth firmly against the upper teeth so as to obtain a good impression; then remove the plate and wax from the mouth and run the model, trim off the surplus wax and invest the case in the flask. When the plaster is hard open the flask and wash out the wax with hot water, cut little retaining pits in the old plate, then pack with rubber and vulcanize as usual. When finished it will make a beautiful piece of work, with very little labor.—*Dr. Gustave North, Cedar Rapids, Iowa.*

---

A Handy Asbestos Block.—I am using an asbestos block of my own make, and as I have found it very useful as well as easy to make, I decided to describe it and enable any dentist who may read this article to give it a trial. It is made in the following manner: Take sufficient asbestos rope, which may be obtained in any hardware store, 36 inches in length will be plenty, and coil it after a manner similar to that in which rope is coiled on board ship, until you have a coil from three to four inches in diameter. Cover this to the thickness of about one inch with a mixture of plaster-of-Paris and pumice, with some threads of asbestos fibre, while the mass is still soft, insert three-inch nails to form an equilateral triangle. When this hardens, you have an asbestos block than can be used on any table without danger of scorching the wood, and also at a very low cost.—*Dr. J. W. Hagey.—Dom. Jour.*

---

Septoforma.—Septoforma is a product of formaldehyde. According to Engels, a three per cent. solution destroys staphyococcus pyogenes aureus in three minutes, the cholera vibrio in one minute, and the typhoid bacillus in ten minutes. Instruments of various metals exposed for three days to thirty per cent. solution of septoforma, and none of them showed the slightest change except those made of aluminum. It seems to have advantages over other similar disinfectants, owing to a much less penetrating odor, absence of local irritation or injury to metal instruments, and that it has a considerable degree of germicidal power. It is recommended for the disinfection of instruments in the strength of five per cent. to ten per cent. solution and as a wash for wounds in a three per cent. solution.—*Dr. Alfonse Irwin, Ex. Items.*

---

Dental Caries, Sodium Hyposulphite in.—The writer recommends a trial of sodium hyposulphite in dental caries accompanied by pulpitis and secretion of pus of a putrid odor and taste. Generally carbolic acid or creosote is used in such cases, but these often produce only little effect, and, besides, they trickle through the dressing and mix with the saliva, to the annoyance of the patient; furthermore, their handling requires great care on account of their causticity. Two cases of dental caries of the nature described were treated by the author with a saturated solution of sodium hyposulphite, applied on cotton and packed into the cavity of the tooth. In a few days the putrid odor and taste in the mouth completely disappeared. In one of the cases carbolic acid had previously been used for quite a while without effecting any appreciable improvement in the condition. Claret (Nouveaux Remedies, xix, No. 3; Merck's Archives, April, 1903).

---

Must Pay the Dentist.—Leavenworth, Kan., Nov. 14.—A jury of twelve ment in the district court held that Dr. C. S. Nichols should have $70 for constructing for Mrs. Mary Jordan, two sets of false teeth. The evidence in the case occupied the entire morning and afternoon sessions of the court and then the jury took charge of it at 4:05 o'clock. At 4:25 o'clock that body returned to the court room with a verdict in favor of Dr.

Nichols, which means that Mrs. Jordan will not only have to pay the $70, but also the cost in the case, the total amount being no less than $125. Dr. Nichols made two sets of false teeth for Mrs. Jordan. It was claimed that she took the first set home and scratched them up with a knife in an attempt to make them fit and then returned them to the dentist, who then made a second set, this set also, it was claimed, Mrs. Jordan tried to fit to her mouth herself, but being unable to do so, returned them to the dentist and refused to pay for either set. Dr. Nichols demanded pay for the teeth; Mrs. Jordan refused. The dentist filed suit in the city court and got a verdict for $38, and Mrs. Jordan took the case to the district court.—*Topeka, Kansas, State Journal.*

---

Asepsis.—The dentist encounters great difficulty in carrying out a perfect asepsis in his instrumentarium, since he operates with so many instruments and changes his patients so often that it is no little matter to sterilize them all before every operation. Instruments sterilized the day before cannot be counted upon as absolutely sterile, on account of the dust of the operating room penetrating into the drawers of the instrument case. I have been able to overcome this difficulty in a very simple manner by placing a formalin tablet in each drawer of my instrument case and renewing it from time to time (about once a week). The drawers remain constantly sterile, and the quantity of formaldehyd vapor which is developed is not sufficient to in any way contaminate the air of the room. Of course the dentist should see to it that the knobs on his instrument case, as well as those parts of his operating chair with which he is obliged to come in contact with his hands during an operation, should be kept in a clean if not in an aseptic condition.—*Dr. W. D. Miller, Ex. Cosmos.*

---

Filling Children's Teeth.—In those cases where the teeth are small, with a tendency to a restricted arch, there is nothing equal to pink base plate gutta-percha for filling badly decayed proximal cavities in deciduous molars. Prepare the cavities as well as possible, dry thoroughly and line with chloro-percha. Then fill with one piece of gutta percha large enough to bridge across and fill both cavities. The force of mastication will constantly wedge the filling against the walls of the cavities, resulting in the development of the alveolar process and jaws so that when the bicuspids are erupted there will be abundance of room for all of the anterior teeth. I have had cases in which the space between the molars was extended from 1-8 to 1-4 of an inch. A piece of German silver wire can be inserted in the filling to protect the tissues in the interproximal space. The sary.—*Dr. C. R. Taylor, Review.*

---

Dentition.—So far as the mouth alone is concerned, a very large majority of the cases of dentin, no matter how much swollen or how painful the gums may be, are simply cases of physiological congestion of the parts involved. The cases where this congestion passes into a real inflammation are rare, and the cases where this inflammation passes on to supperation are rarer still. So that in a very large majority of the cases there is nothing local in the mouth to cause fever, and if fever exists we should look elsewhere for the cause. Uncomplicated cases of dentition the physician does not see. It is only when fever exists that his services are in demand; but the mother naturally looks to the mouth as the seat of the trouble, and the physician, unless he stops and considers, looks to the mouth, too. But the case now ceases to be one of dentition, becoming one of something else occurring during dentition, and far removed from the mouth.—*Dr. E. R. Corson, Ex. Era.*

---

Method of Treating a Chronic Abscess.—A chronic alveolar abscess with fistulous opening, may be cured in one treatment (provided a fairly good communication can be established through the pulp canal and out the fistula) as follows: Open up pulp chamber freely, remove all debris possible from root canal and irrigate thoroughly with your favorite antiseptic wash, being careful to force the liquid through the canal and fistula. Dry the canal as thoroughly as possible, wipe out with carbolic acid, creosote or oil of cloves, and pump chloro-percha through the canal until it appears at the fistula. Insert your gutta-percha canal point and also the permanent filling in the crown, and you can dismiss the patient with the assurance that the fistula will heal from the bottom outward and disappear entirely in a few days. I have never had this treatment fail. *Dr. Arthur G. Smith, Review.*

---

Thoroughness.—The plea for thoroughness cannot be too strongly emphasized, because thoroughness is the keynote to all success. If the root-canals of a devitalized tooth be not thoroughly filled, no matter how carefully manipulated the superstructure (whether filling or crown) may be, a failure will ensue. If the caries be not thoroughly removed and the cavity extended and shaped so that caries will not recur and anchorage be secure, a failure will ensue. If the filling be not properly trimmed so as to free the gingivae, more or less discomfort and an unhealthy condition of the gum will result.

If not properly contoured, food will crowd down against the gum, causing it to recede, and in addition to that the particles of food so lodged will disintegrate and caries take place beyond the gingival margin of the filling. With a proper contour and the filling extending under a healthy gum, caries cannot take place. It pays to be thorough.—*Dr. J. M. Magee, Brief.*

---

Capping Pulps.—The capping of pulps other than those exposed mechanically is rarely attended with any degree of success; that is to say, though a pulp may become quiet for a time, dissolution almost invariably takes place. Instead of capping a pulp exposed by caries, which always leaves it in a more or less morbid condition, it can be treated with a mummifying agent and assured a longer period of comfort for the tooth. I believe, however, that it is necessary in using a drug of this character to have the pulp thoroughly inoculated with it. This is best accomplished by puncturing the pulp with a sharp probe, causing it to bleed, and forcing the drug in under a gentle but firm pressure, in the same manner that pressure anesthesia is applied. I do not wish to be understood as advocating the mummification of pulps indiscriminately. My experience with this method covers a period of but two years, and has been confined to cases where either devitalization or capping of the pulp was indicated.

I would say that wherever the importance of preserving the vitality of the pulp is recognized, such methods should be adopted as will be in conformity with the various abnormal conditions present, and that any operation which might jeopardize its wellbeing should be avoided.—*Dr. J. C. Salvas, Ex. Brief.*

